# Evaluating Pressure Variability and Influencing Factors during High-Flow Nasal Cannula Therapy in Premature Infants

**DOI:** 10.3390/children11080995

**Published:** 2024-08-15

**Authors:** Fong-Cheng Ho, Chia-Ying Lin, Ane-Shu Chang, Ching-Yi Yeh, Hsiu-Lin Chen

**Affiliations:** 1 Division of Neonatology, Department of Pediatrics, Kaohsiung Medical University Hospital, Kaohsiung Medical University, Kaohsiung 807, Taiwan; 1030315@kmuh.org.tw; 2 Department of Pediatrics, Kaohsiung Municipal Ta-Tung Hospital, Kaohsiung 801, Taiwan; 3 Graduate Institute of Medicine, College of Medicine, Kaohsiung Medical University, Kaohsiung 807, Taiwan; 1050181@kmuh.org.tw; 4 Division of Respiratory Therapy, Kaohsiung Medical University Hospital, Kaohsiung 807, Taiwan; 980151@kmuh.org.tw (A.-S.C.); 900237@kmuh.org.tw (C.-Y.Y.); 5 Department of Respiratory Therapy, College of Medicine, Kaohsiung Medical University, Kaohsiung 807, Taiwan

**Keywords:** premature infants, high-flow nasal cannula, continuous positive airway pressure, noninvasive respiratory support

## Abstract

Background: Heated humidified high-flow nasal cannulas (HHHFNCs) are increasingly used as an alternative strategy for weaning from nasal continuous positive airway pressure (NCPAP) in premature infants. However, the optimal pressure provided by HHHFNCs is unknown. This retrospective study investigated the pressure changes and associated factors during HHHFNC therapy in preterm infants. Methods: Clinically stable preterm neonates born with a birth weight of 2500 g or less and receiving HHHFNC therapy for weaning from NCPAP were enrolled. The flow of the HHHNFCs was adjusted to achieve an initial pressure equivalent to the positive expiratory pressure (PEEP) of NCPAP. Subsequent pressure changes in the HHHFNCs were measured by a GiO digital pressure gauge. Results: Nine premature infants were enrolled. Their gestational age (mean ± SD) was 28.33 ± 2.61 weeks, and the birth weight was 1102.00 ± 327.53 g. Overall, 437 pressure measurements were conducted. The median pressure of the HHHFNCs was 5 cmH_2_O. The generated pressure had a significant association with the body weight, postmenstrual age (PMA) and flow rate. A multiple regression model revealed that the measured pressure (cmH_2_O) = −5.769 + 1.021 × flow rate (L/min) − 0.797 × body weight (kg) + 0.035 × PMA (days) (r^2^ = 0.37, *p* < 0.001). Conclusions: The pressure provided by HHHFNCs is influenced by body weight, PMA, and flow rate. It is feasible to set the delivered pressure of HHHFNCs to match the applied PEEP of NCPAP initially, facilitating the weaning of preterm infants from NCPAP to HHHFNCs.

## 1. Introduction

Heated humidified high-flow nasal cannulas (HHHFNCs) have been introduced into neonatal respiratory care as a means of providing noninvasive respiratory support for neonates with respiratory distress syndrome (RDS) and apnea of prematurity (AOP), and to assist premature infants post-extubation as an alternative to nasal continuous positive airway pressure (NCPAP) [1,2]. HHHFNCs improved patient tolerance by delivering heated, humidified, and blended oxygen through a nasal cannula at flow rates exceeding 2 L/min [3]. This technique can enhance alveolar ventilation and end-expiratory lung volume, improve lung compliance, reduce nasopharyngeal dead space and decrease the risk of ventilator-induced lung injury [4,5,6]. For several years, HHHFNCs have emerged as a popular alternative to NCPAP for respiratory support in pediatric care [7]. Both HHHFNCs and NCPAP are noninvasive methods that provide continuous positive airway pressure to spontaneously breathing patients and are effective in preventing extubation failure [1,8]. However, NCPAP is associated with several complications, including traumatic injuries to the nose, pulmonary air leak syndromes, and gaseous distension of the stomach [9]. Osman et al. assessed pain in preterm infants using both HHHFNCs and NCPAP and found that infants in the HHHFNC group experienced significantly less pain and showed improved tolerance [10]. Due to its small tapered prongs and simple interface, HHHFNCs are easier for nursing staff to use [11] and more conducive to mother–infant bonding compared to NCPAP [12]. Additionally, previous studies have found that oral feeding while on HHHFNC therapy is safe and well tolerated [13,14].

Despite the many documented benefits of HHHFNCs, the optimal maximal flow rate for HHHFNC therapy remains unclear. The circuit flow can be adjusted based on clinical parameters such as the patient’s age and weight, the desired level of positive end-expiratory pressure (PEEP), and the experience of healthcare providers [15,16]. However, the pressures generated are not routinely measured in clinical practice, and it is unknown what liters per minute of gas flow should be applied with the HHHFNC system to achieve the desired distending positive pressure. Infants receiving HHHFNC therapy with excessive pressure delivery to their airway may experience significant lung overexpansion and are at risk of severe side effects [17,18].

To date, few studies have investigated the pressure generated by HHHFNCs when monitored at the bedside. Therefore, this study aims to examine the changes in bedside pressure and the factors influencing these changes during HHHFNC therapy among preterm infants who use HHHFNC devices as an alternative to NCPAP.

## 2. Materials and Methods

### 2.1. Patients

This retrospective observational study was conducted from March 2015 to August 2015 in the neonatal intensive care unit (NICU) at a hospital in Taiwan. Preterm neonates with a body weight of 2500 g or less, receiving HHHFNC ventilator support as an alternative to NCPAP, were enrolled. Infants with congenital anomalies, cyanotic heart disease, or neuromuscular disorder were excluded. This study received approval from the Institutional Review Board Committee of the Kaohsiung Medical University (approval number: KMUHIRB-SV(I)-20200028). Since this study was conducted as a retrospective review of medical records, the Institutional Review Board (IRB) did not require individual parental consent.

### 2.2. Pressure Measurements during HHHFNC Therapy

**Figure 1:**
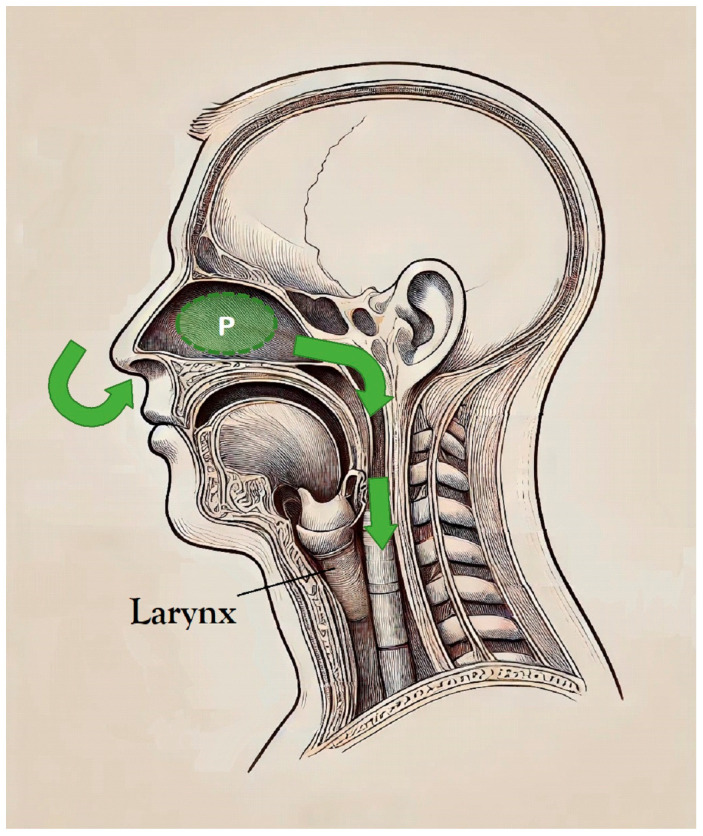
Illustration of the pressure transmission pathway in high-flow nasal cannula therapy, depicting the flow from the nasal cannula through the nasopharynx, oropharynx, larynx, and into the trachea. P, pressure.

**Figure 2:**
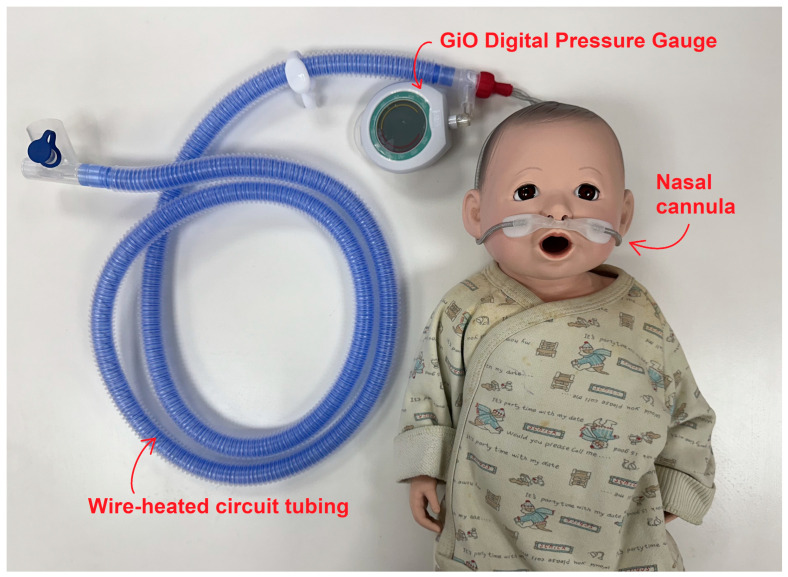
Detailed view of the GiO digital pressure gauge connected to the proximal circuit of the high-flow nasal cannula setup, highlighting how the device measures pressure.

The pressure transmission pathway in high-flow nasal cannula therapy is illustrated in Figure 1, which details the route through which pressure is conveyed by the high-flow nasal cannula. This illustration tracks the pressure journey from the nasopharynx through the oropharynx, larynx, and finally the trachea. We postulate that the pressure in the nasopharynx is directly influenced by the flow from the nasal cannula, with the most proximal measurements reflective of this effect. To accurately capture this pressure, we utilized the GiO digital pressure gauge connected to the proximal circuit of the high-flow nasal cannula setup, as depicted in Figure 2. The GiO digital pressure gauge (GIO 6, GaleMed Corporation, Taipei, Taiwan) is a portable, noninvasive, and cost-effective device that displays real-time pressure measurements. It is capable of measuring pressures ranging from −250 cmH_2_O to +250 cmH_2_O. In our study, it was used to monitor the pressure at the distal end of the nasal cannula (illustrated in Figure 2). This method of using the GiO gauge to monitor HHHFNCs provides a direct, real-time assessment of the pressures being delivered to the patient, aiding in the precise adjustment of flow rates to avoid excessive lung-distending pressures, especially critical in preterm neonates. While measuring the pressure, we attempted to minimize the pressure release effect by keeping the infants’ mouths closed and used prongs with diameters based on the manufacturer’s guidelines. This setup allows us to closely monitor and estimate the pressure dynamics within the nasopharynx, thus providing insights into the overall airway pressure. Enrolled infants, deemed stable by the clinical team, received respiratory support using the HHHFNC Optiflow™ junior device with the MR 850 humidifier and binasal infant cannula (Fisher & Paykel Opti flow System, Healthcare, Auckland, New Zealand). Each infant was initially started on HHHNFC therapy with a flow rate adjusted to achieve a generated pressure equal to the positive expiratory pressure (PEEP) of NCPAP. The generated pressure at the distal end of the nasal cannula was measured using a GiO digital pressure gauge. This pressure was documented twice daily until HHHFNC therapy was discontinued.

### 2.3. Covariates and Outcome Measures

Neonatal variables collected included gestational age, birth weight, sex, 1 min and 5 min Apgar score. Additional data such as body weight, postmenstrual age (PMA) prior to initiating HHHFNC therapy, and the flow rate and pressure provided during HHHFNC therapy were gathered for analysis. The primary outcome was the changes in pressure during HHHFNC therapy. Secondary outcomes included potential influencing factors such as body weight, PMA, and flow rate of HHHFNC.

### 2.4. Statistical Analysis

Data are presented as mean ± SD and as percentages. To explore the association between potential influencing factors and the pressure generated by the HHHFNC, linear regression analyses were conducted, with the generated pressure as the dependent variable and the patient’s body weight, PMA, and HHHFNC flow rate as the independent variables. A multiple regression analysis was also performed to examine the relationship between generated pressure and these three variables. *p*-values less than 0.05 were considered statistically significant. All analyses were carried out using IBM SPSS Statistics V22.0 (SPSS Inc., IBM Corp., Armonk, NY, USA).

## 3. Results

### 3.1. Participants

**Figure 3:**
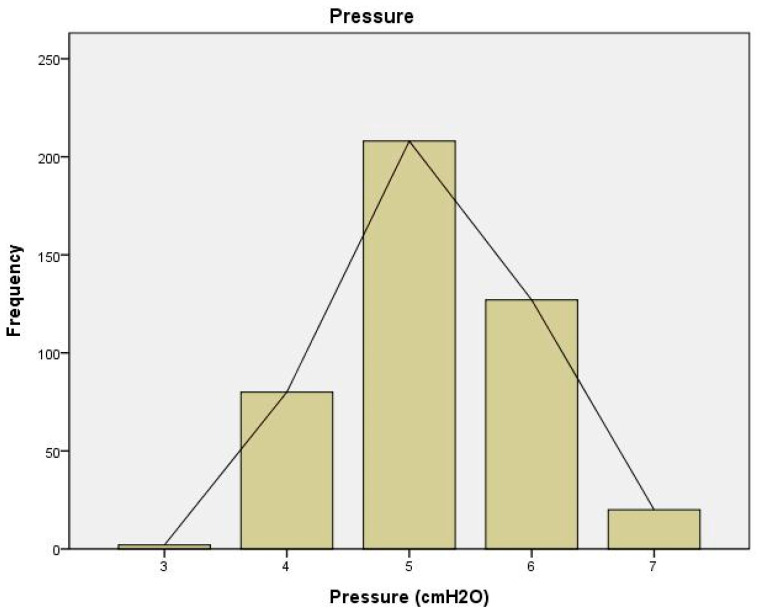
Frequency distribution of the pressures measured by high-flow high-humidity nasal cannulas (HHHFNCs) in study infants, illustrating the variability of pressure levels.

Nine premature infants (four males and five females) were enrolled in this study. Among the preterm infants enrolled, five were diagnosed with bronchopulmonary dysplasia (BPD) at the time of their inclusion—one case was classified as moderate, and four as severe. Table 1 provides details on the characteristics of the participants and baseline respiratory support settings. During the study, 437 different pressure measurements were conducted while using the HHHFNCs. During the study measurements, the median flow rate was 4 L/min, and the median pressure provided by the HHHFNCs was 5 cmH_2_O. The distribution of pressure generated by the HHHFNCs is shown in Figure 3.

### 3.2. Analysis of Factors That Affect Generated Pressure of HHHFNCs

**Figure 4:**
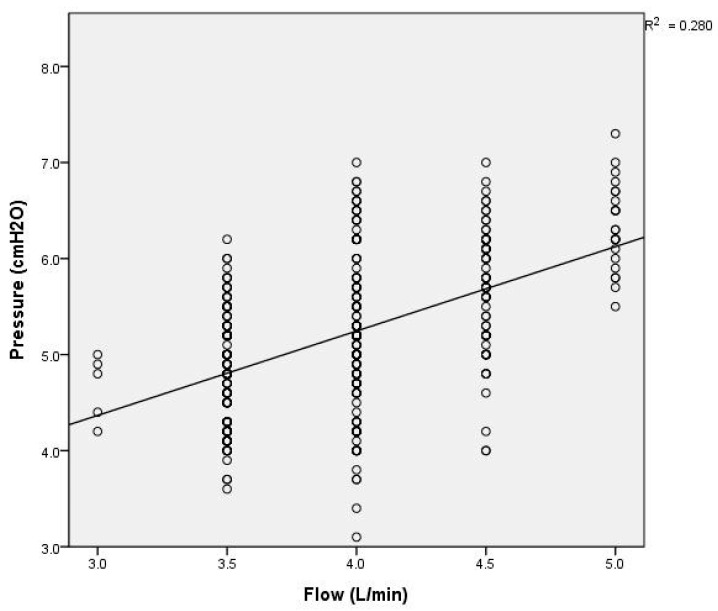
Scatter plot depicting the relationship between flow rate and pressure measured by HHHFNCs, with trend lines indicating correlations.

**Figure 5:**
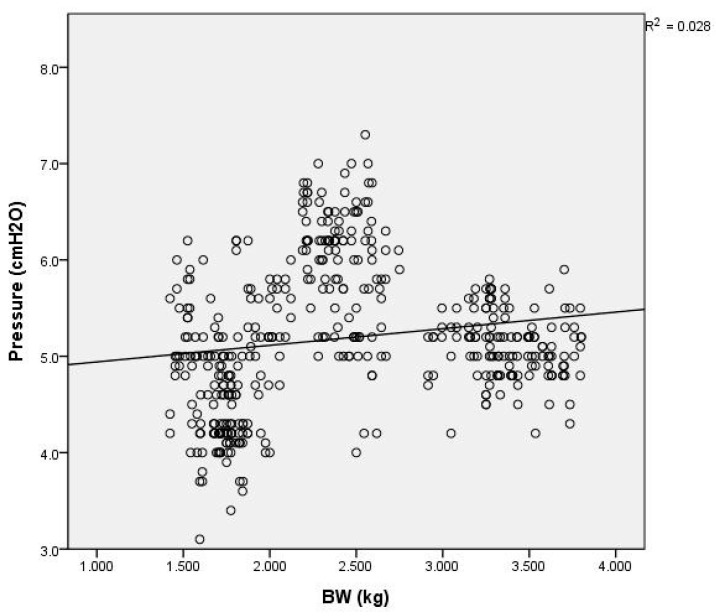
Scatter plot showing the relationship between body weight and pressure measured by HHHFNCs, with analysis of variance impacts.

**Figure 6:**
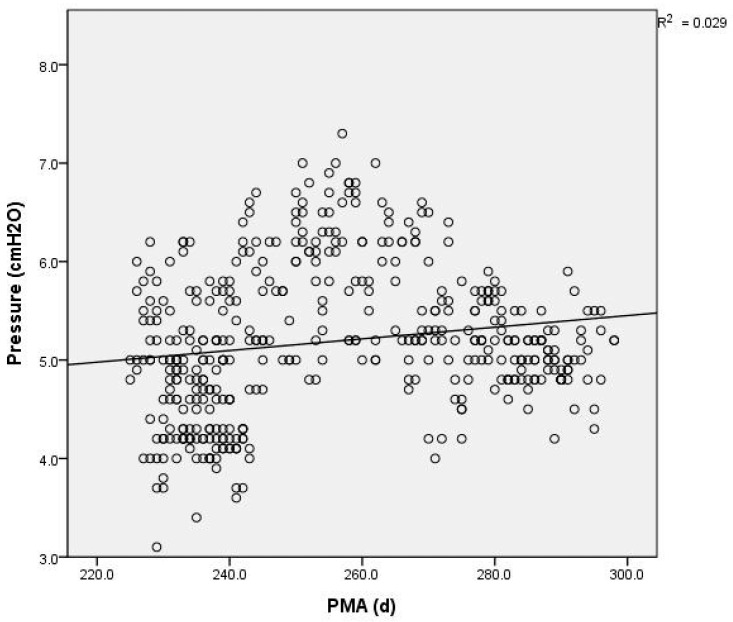
Scatter plot of the relationship between postmenstrual age and pressure measured by HHHFNCs, assessing developmental influences on pressure efficacy.

The generated pressure was significantly influenced by the flow rates, showing a notable increase (r^2^ = 0.28, *p* < 0.0001) at higher flows (see Figure 4). Additionally, weight was positively correlated with pressure (r^2^ = 0.028, *p* < 0.0001), indicating that the pressure provided by the HHHFNCs was higher in the larger infants (see Figure 5). There was also a positive correlation (r^2^ = 0.029, *p* < 0.0001) between the generated pressure and PMA. The pressure of the HHHFNCs was statistically significantly higher with advancing PMA (see Figure 6).

To further investigate the stability of the HHHFNC pressure, we have calculated the coefficient of variation of pressure for each infant involved in the study. The results, presented in Table 2, show individual coefficients of variation for each monitored pressure instance. Notably, an increase in this coefficient could signify instability in HHHFNC pressure delivery, although it is important to recognize that such variations could also stem from other influencing factors. To account for factors related to the nasal cavity, we have incorporated PMA and body weight as indicators of space in the nasopharynx. Subsequently, we conducted multiple linear regression analyses to examine these factors comprehensively.

Multiple linear regression analyses revealed that the flow rate, current weight, and PMA significantly predicted the pressure generated by the HHHFNCs. The flow rate was the most significant independent variable, followed by the current weight and PMA. The predicted generated pressure can be calculated using the following formula: −5.769 + 1.021 × flow rate (L/min) − 0.797 × body weight (kg) + 0.035 × PMA (days) (r^2^ = 0.37, *p* < 0.001). In this equation, the weight is negatively influenced by the generated pressure.

### 3.3. Adverse Events

Adverse events, including air leaks (such as pneumothorax and pneumomediastinum), facial/nasal injuries, or gastrointestinal perforations, were not reported among the enrolled infants during the course of the HHHFNC therapy.

## 4. Discussion

In this study, we examined nine premature infants through 437 distinct pressure measurements generated by HHHFNCs. Our findings indicate that the pressure delivery was inversely related to the infant weight and directly proportional to both the HHHFNC flow rate and PMA. Furthermore, we demonstrated that bedside pressure measurements using a GiO digital pressure gauge are feasible. This capability may assist clinicians in adjusting the HHHFNC flow rate to prevent the inadvertent delivery of excessive lung-distending pressure, particularly in preterm neonates.

HHHFNC therapy has seen increasingly use in recent years. Previous studies have indicated that HHHFNCs are as effective as NCPAP in preventing extubation failure and managing apnea of prematurity [19,20]. This device can also serve as an alternative to NCPAP for respiratory support in pediatric care [7]. However, there is currently no standard guideline for the optimal setting while transitioning from NCPAP to HHHFNCs. Our findings confirm that the initial pressure delivered by HHHFNCs can be set to match the applied PEEP of NCPAP without resulting in adverse effects, such as pneumothorax, pneumomediastinum, or gastrointestinal perforations.

The pressure generated by high-flow nasal cannulas (HFNCs) has been the subject of prior studies. An in vitro study demonstrated that pressure delivery is directly proportional to the HFNC flow and the nasal prong-to-nares ratio [21]. Wilkinson et al. found that the pharyngeal pressure in 18 preterm infants receiving HFNC therapy was associated with higher flow rates and lower infant weights [22]. Similarly, our study showed that the HHHFNC pressure increased linearly with the flow delivered (see Figure 4).

However, it is not expected to find a positive correlation between the HHHFNC’s generated pressure and the infant weight. Generally, as infants gain weight, they tend to have relatively larger nostrils and increased nasopharyngeal dead spaces, which may increase the work of breathing and reduce respiratory efficiency. Based on the above analysis of factors influencing the pressure generated by HHHFNCs, we have found that the relationships may not be strictly linear, despite the low *p*-values previously indicated. This realization prompted us to consider additional variables that could affect pressure measurements, such as variations in the nasal cavity space and the impact of forced respiration. To assess the stability of HHHFNC pressure delivery, we calculated the coefficient of variation of pressure for each infant in the study. The findings, detailed in Table 2, reveal the individual coefficients of variation for each instance of pressure monitoring. An increase in this coefficient may suggest potential instability in HHHFNC pressure delivery, although it is crucial to note that these fluctuations may also arise from other factors. Our results showed that the nine infants examined had median coefficients of variation ranging from 6.8% to 16.1%. These figures generally indicate stable pressure support from HHHFNCs in low-birth-weight premature infants. To further explore factors related to the nasal cavity, we included the postmenstrual age (PMA) and body weight as indicators of the nasopharyngeal space. Therefore, we analyzed the associated factors using multiple regression analyses. There was a significant correlation between the variables, as shown by the regression equation for predicted pressure generated by HHHFNCs: −5.769 + 1.021 × flow rate (L/min) − 0.797 × body weight (kg) + 0.035 × PMA (days). This equation reveals an inverse relationship between the HHHFNC pressure and infant weight, aligning with findings from previous studies [22,23,24]. Clinicians should be cautious of the pressures delivered to vulnerable preterm neonates when setting flow rates.

The generated pressure can be influenced by several factors, including increased flow rate, the opening of the mouth (which reduces pressure), the infant’s weight (which correlates negatively), and the outer diameter of the prongs (where a larger diameter leads to higher occlusion rates and consequently higher pressures) [24,25]. In our study, we attempted to minimize the pressure release effect by keeping the infants’ mouths closed and used prongs with diameters based on the manufacturer’s guidelines. However, our discussions led us to consider the nasopharyngeal space as a potential influencing factor. This insight prompted the inclusion of PMA in our analysis to further understand its impact on pressure dynamics.

The main limitation of this study is the small sample size. Although HHHFNCs have been used in our NCIU since 2015, the number of cases has remained low due to the requirement for self-payment. To mitigate the effects of inter-patient variation, we collected 437 different pressure measurements generated by the HHHFNCs. Furthermore, the effect of leaks at the mouth and nasal interface was not assessed in our study. We attempted to standardize the amount of leaks by passively closing the mouth during the study procedures. Nevertheless, our observations demonstrated a linear trend of increasing HHHFNC-generated pressure with decreasing body weight, increasing HHHFNC flow, and advancing PMA. Larger trials, adequately powered to ascertain clinically relevant outcomes and to assess the associated factors influencing HHHFNC-generated pressure, are necessary.

## 5. Conclusions

In summary, the HHHFNC pressure is influenced by body weight, PMA, and flow rate. Our analysis reveals that these relationships are not strictly linear. We observed variability in HHHFNC pressure delivery, as indicated by the coefficient of variation for each infant, which may be influenced by factors such as the nasopharyngeal space. From a clinical perspective, these findings are significant as they suggest that adjustments in HHHFNC settings need to be carefully considered based on individual patient characteristics, especially the PMA and body weight. This can aid in optimizing the therapeutic efficacy of HHHFNCs, minimizing potential complications associated with inappropriate pressure settings. Furthermore, our findings confirm that it is feasible to initially set the delivered pressure of HHHFNCs to match the applied PEEP of NCPAP when transitioning preterm infants from NCPAP to HHHFNCs.

## Figures and Tables

**Table 1:** Characteristics and baseline respiratory support settings of HHHFNCs among enrolled infants.

	Mean	Median
Birth gestation (weeks)	28.33 ± 2.61	28.4
Birth weight (g)	1102 ± 327.53	1153
1 min Apgar score	3.89 ± 1.76	4
5 min Apgar score	6 ± 2.24	6
Respiratory support with HHHFNCs		
PMA (weeks)	46.67 ± 26.83	30
PMA (days)	244.44 ± 19.74	238
Current weight (g)	2083 ± 11	1917
Flow (L/min)	3.83 ± 0.56	4
Pressure (cmH_2_O)	4.89 ± 0.81	5
Duration (days)	20.89 ± 4.73	21

HHHFNC, heated humidified high-flow nasal cannula; PMA, postmenstrual age.

**Table 2:** Individual coefficient of variation of the pressure.

Participant	Mean	Median	Std. Deviation	Coefficient of Variation	
				Mean Centered	Median Centered
1	4.995	5.000	0.338	6.8%	6.8%
2	2.450	2.450	0.316	13.2%	13.1%
3	1.333	1.333	0.168	11.5%	16.1%
4	1.250	1.250	0.169	13.5%	13.5%
5	0.667	0.667	0.087	12.2%	14.8%
6	0.857	0.857	0.081	9.3%	9.7%
7	0.638	0.625	0.033	5.2%	5.7%
8	0.667	0.667	0.080	12.6%	12.5%
9	0.518	0.518	0.060	11.9%	12.0%

## Data Availability

All data generated or analyzed during this study are included in the published article.
